# Exploring factors influencing domestic violence: a comprehensive study on intrafamily dynamics

**DOI:** 10.3389/fpsyt.2023.1243558

**Published:** 2023-09-07

**Authors:** Cintya Lanchimba, Juan Pablo Díaz-Sánchez, Franklin Velasco

**Affiliations:** ^1^Departamento de Economía Cuantitativa, Facultad de Ciencias Escuela Politécnica Nacional, Quito, Ecuador; ^2^Institut de Recherche en Gestion et Economie, Université de Savoie Mont Blanc (IREGE/IAE Savoie Mont Blanc), Annecy, France; ^3^Department of Marketing, Universidad San Francisco de Quito USFQ, Quito, Ecuador

**Keywords:** domestic violence, depression, mood, religious affiliation, health consciousness, quantile regression, Ecuador, Covid-19

## Abstract

**Introduction:**

This econometric analysis investigates the nexus between household factors and domestic violence. By considering diverse variables encompassing mood, depression, health consciousness, social media engagement, household chores, density, and religious affiliation, the study aims to comprehend the underlying dynamics influencing domestic violence.

**Methods:**

Employing econometric techniques, this study examined a range of household-related variables for their potential associations with levels of violence within households. Data on mood, depression, health consciousness, social media usage, household chores, density, and religious affiliation were collected and subjected to rigorous statistical analysis.

**Results:**

The findings of this study unveil notable relationships between the aforementioned variables and levels of violence within households. Positive mood emerges as a mitigating factor, displaying a negative correlation with violence. Conversely, depression positively correlates with violence, indicating an elevated propensity for conflict. Increased health consciousness is linked with diminished violence, while engagement with social media demonstrates a moderating influence. Reduction in the time allocated to household chores corresponds with lower violence levels. Household density, however, exhibits a positive association with violence. The effects of religious affiliation on violence manifest diversely, contingent upon household position and gender.

**Discussion:**

The outcomes of this research offer critical insights for policymakers and practitioners working on formulating strategies for preventing and intervening in instances of domestic violence. The findings emphasize the importance of considering various household factors when designing effective interventions. Strategies to bolster positive mood, alleviate depression, encourage health consciousness, and regulate social media use could potentially contribute to reducing domestic violence. Additionally, the nuanced role of religious affiliation underscores the need for tailored approaches based on household dynamics, positioning, and gender.

## 1. Introduction

Intimate partner violence is a pervasive global issue, particularly affecting women. According to the World Health Organization (1), approximately 30% of women worldwide have experienced violence from their intimate partners. Disturbingly, recent studies indicate that circumstances such as the COVID-19 pandemic, which disrupt daily lives on a global scale, have exacerbated patterns of violence against women (2–4). Data from the WHO (1) regarding gender-based violence during the pandemic reveals that one in three women felt insecure within their homes due to family conflicts with their partners.

This pressing issue of intimate partner violence demands a thorough analysis from a social perspective. It is often insidious and challenging to identify, as cultural practices and the normalization of abusive behaviors, such as physical aggression and verbal abuse, persist across diverse socioeconomic backgrounds. However, all forms of violence can inflict physical and psychological harm on victims, affecting their overall well-being and interpersonal relationships WHO (5). Furthermore, households with a prevalence of domestic violence are more likely to experience child maltreatment (6).

In this context, the COVID-19 pandemic has had profound effects on individuals, families, and communities worldwide, creating a complex landscape of challenges and disruptions. Among the numerous repercussions, the pandemic has exposed and exacerbated issues of domestic violence within households. The confinement measures, economic strain, and heightened stress levels resulting from the pandemic have contributed to a volatile environment where violence can escalate. Understanding the factors that influence domestic violence during this unprecedented crisis is crucial for developing effective prevention and intervention strategies.

This article aims to explore the relationship between household factors and domestic violence within the context of the COVID-19 pandemic. By employing econometric analysis, we investigate how various factors such as mood, depression, health consciousness, social media usage, household chores, density, and religious affiliation relate to violence levels within households. These factors were selected based on their relevance to the unique circumstances and challenges presented by the pandemic.

The study builds upon existing research that has demonstrated the influence of individual and household characteristics on domestic violence. However, the specific context of the pandemic necessitates a deeper examination of these factors and their implications for violence within households. By focusing on variables that are particularly relevant in the crisis, we aim to provide a comprehensive understanding of the dynamics that contribute to intrafamily violence during the pandemic.

The findings of this study have important implications for policymakers, practitioners, and researchers involved in addressing domestic violence. By identifying the factors that either increase or mitigate violence within households, we can develop targeted interventions and support systems to effectively respond to the unique challenges posed by the pandemic. Furthermore, this research contributes to the broader literature on domestic violence by highlighting the distinct influence of household factors within the context of a global health crisis.

The structure of this paper is organized as follows. Section 2 provides a comprehensive review of the relevant literature on household violence. Section 3 presents the case study that forms the basis of this research. Section 4 outlines the methodology employed in the study. Section 5 presents the results obtained from the empirical analysis. Finally, Section 6 concludes the paper, summarizing the key findings and their implications for addressing domestic violence.

## 2. Literature review

### 2.1. Violence at home

Throughout human history, the family unit has been recognized as the fundamental building block of society. Families are comprised of individuals bound by blood or marriage, and they are ideally regarded as havens of love, care, affection, and personal growth, where individuals should feel secure and protected. Unfortunately, it is distressingly common to find alarming levels of violence, abuse, and aggression within the confines of the home (7).

Domestic violence, as defined by Tan and Haining (8), encompasses any form of violent behavior directed toward family members, regardless of their gender, resulting in physical, sexual, or psychological harm. It includes acts of threats, coercion, and the deprivation of liberty. This pervasive issue is recognized as a public health problem that affects all nations. It is important to distinguish between domestic violence (DV) and intimate partner violence (IPV), as they are related yet distinct phenomena. DV occurs within the family unit, affecting both parents and children. On the other hand, IPV refers to violent and controlling acts perpetrated by one partner against another, encompassing physical aggression (such as hitting, kicking, and beating), sexual, economic, verbal, or emotional harm (9, 10). IPV can occur between partners who cohabit or not, and typically involves male partners exerting power and control over their female counterparts. However, it is crucial to acknowledge that there are cases where men are also victims of violence (11).

Both forms of violence, DV and IPV, take place within the home. However, when acts of violence occur in the presence of children, regardless of whether they directly experience physical harm or simply witness the violence, the consequences can be profoundly detrimental (12, 13).

Understanding the intricacies and dynamics of domestic violence and its impact on individuals and families is of paramount importance. The consequences of such violence extend beyond the immediate victims, affecting the overall well-being and social fabric of society. Therefore, it is crucial to explore the various factors that contribute to domestic violence, including those specific to the current context of the COVID-19 pandemic, in order to inform effective prevention and intervention strategies. In the following sections, we will examine the empirical findings regarding household factors and their association with domestic violence, shedding light on the complexities and nuances of this pervasive issue.

### 2.2. Drivers of domestic violence

As previously discussed, the occurrence of violence within the home carries significant consequences for individuals’ lives. Consequently, gaining an understanding of the underlying factors that contribute to this violence is crucial. To this end, Table 1 provides a comprehensive summary of the most commonly identified determinants of domestic violence within the existing literature.

**Table 1:** Determinants of domestic violence.

Determinant	Referred study
(A) Demographic characteristics	
(A1) Education of the head of household and of the woman	Erten and Keskin (14), Krob and Steffen (15), and Visaria (16)
(A2) Employment and occupation	Alonso-Borrego and Carrasco (17), Anderberg et al. (18), Sen (19), and Visaria (16)
(A3) Religion	Krob and Steffen (15), Tomisin (20), Visaria (16), and Zeybek and Arslan (21)
(B) Presence of a risk factor	
(B1) Health – psychological problems (Depression, anxiety and stress)	Van de Velde et al. (22), Straus et al. (23), Burney (24), Cooper and Smith (25), Heise and Garcia-Moreno (26), Langford et al. (27), Walker-Descartes et al. (28), and WHO (5)
(B2) Retention Tendency	Ishola (29)
(B3) Density	Barrientos et al. (30)
(B4) Reason for confrontation (divorce, jealousy).	Burney (24), Fareo (31), Heise and Garcia-Moreno (26), and WHO (5)

Adapted and improved from the classification proposed by Visaria (16).

Identifying these determinants is a vital step toward comprehending the complex nature of domestic violence. By synthesizing the findings from numerous studies, Table 1 presents a consolidated overview of the factors that have been consistently associated with domestic violence. This compilation serves as a valuable resource for researchers, practitioners, and policymakers seeking to address and mitigate the prevalence of domestic violence.

The determinants presented in Table 1 encompass various variables, including socio-economic factors, mental health indicators, interpersonal dynamics, and other relevant aspects. By examining and analyzing these determinants, researchers have made significant progress in uncovering the underlying causes and risk factors associated with domestic violence.

It is important to note that the determinants listed in Table 1 represent recurring themes in the literature and are not an exhaustive representation of all potential factors influencing domestic violence. The complex nature of this issue necessitates ongoing research and exploration to deepen our understanding of the multifaceted dynamics at play. Thus, we categorize these factors into two groups to provide a comprehensive understanding of the issue.

Group A focuses on variables that characterize both the victim and the aggressor, which may act as potential deterrents against femicide. Previous research by Alonso-Borrego and Carrasco (17), Anderberg et al. (18), Sen (19), and Visaria (16) has highlighted the significance of factors such as age, level of education, employment status, occupation, and religious affiliation. These individual characteristics play a role in shaping the dynamics of domestic violence and can influence the likelihood of its occurrence.

Group B aims to capture risk factors that contribute to the presence of violence within the home. One prominent risk factor is overcrowding, which can lead to psychological, social, and economic problems within the family, ultimately affecting the health of its members. Research by Van de Velde et al. (21), Walker-Descartes et al. (23), Malik and Naeem (2) supports the notion that individuals experiencing such distress may resort to exerting force or violence on other family members as a means of releasing their frustration. Additionally, Goodman (32) have highlighted the increased risk of violence in households with multiple occupants, particularly in cases where individuals are confined to a single bedroom. These concepts can be further explored through variables related to health, depression, anxiety, and stress, providing valuable insights into the mechanisms underlying domestic violence.

By investigating these factors, our study enhances the existing understanding of the complex dynamics of domestic violence within the unique context of the pandemic. The COVID-19 crisis has exacerbated various stressors and challenges within households, potentially intensifying the risk of violence. Understanding the interplay between these factors and domestic violence is essential for the development of targeted interventions and support systems to mitigate violence and its consequences.

### 2.3. Demographic characteristics (A)

#### 2.3.1. Education level (A1)

According to Sen (19), the education level of the victim, typically women, or the head of household is a significant antecedent of domestic violence. Women’s access to and completion of secondary education play a crucial role in enhancing their capacity and control over their lives. Higher levels of education not only foster confidence and self-esteem but also empower women to seek help and resources, ultimately reducing their tolerance for domestic violence. Babu and Kar (33), Semahegn and Mengistie (34) support this perspective by demonstrating that women with lower levels of education and limited work opportunities are more vulnerable to experiencing violence.

When women assume the role of the head of the household, the likelihood of violence within the household, whether domestic or intimate partner violence, increases significantly. This has severe physical and mental health implications for both the woman and other family members, and in the worst-case scenario, it can result in the tragic loss of life (22, 23, 35).

Conversely, men’s economic frustration or their inability to fulfill the societal expectation of being the “head of household” is also a prominent factor contributing to the perpetration of physical and sexual violence within the home (36).The frustration arising from economic difficulties, combined with the frequent use of drugs and alcohol, exacerbates the likelihood of violent behavior.

These findings underscore the importance of addressing socio-economic disparities and promoting gender equality in preventing and combating domestic violence. By enhancing women’s access to education, improving economic opportunities, and challenging traditional gender roles, we can create a more equitable and violence-free society. Additionally, interventions targeting men’s economic empowerment and addressing substance abuse issues can play a pivotal role in reducing violence within the home.

#### 2.3.2. Employment and occupation (A2)

Macroeconomic conditions, specifically differences in unemployment rates between men and women, have been found to impact domestic violence. Research suggests that an increase of 1% in the male unemployment rate is associated with an increase in physical violence within the home, while an increase in the female unemployment rate is linked to a reduction in violence (37).

Moreover, various studies (34, 35, 38, 39) have highlighted the relationship between domestic violence and the husband’s working conditions, such as workload and job quality, as well as the income he earns. The exercise of authority within the household and the use of substances that alter behavior are also associated with domestic violence.

Within this context, economic gender-based violence is a prevalent but lesser-known form of violence compared to physical or sexual violence. It involves exerting unacceptable economic control over a partner, such as allocating limited funds for expenses or preventing them from working to maintain economic dependence. This form of violence can also manifest through excessive and unsustainable spending without consulting the partner. Economic gender-based violence is often a “silent” form of violence, making it more challenging to detect and prove (40).

Empowerment becomes a gender challenge that can lead to increased violence, as men may experience psychological stress when faced with the idea of women earning more than them (14, 18). Lastly, Alonso-Borrego and Carrasco (17) and Tur-Prats (41) conclude that intrafamily violence decreases only when the woman’s partner is also employed, highlighting the significance of economic factors in influencing domestic violence dynamics.

Understanding the interplay between macroeconomic conditions, employment, and economic control within intimate relationships is crucial for developing effective interventions and policies aimed at reducing domestic violence. By addressing the underlying economic inequalities and promoting gender equality in both the labor market and household dynamics, we can work toward creating safer and more equitable environments that contribute to the prevention of domestic violence.

#### 2.3.3. Religion (A3)

Religion and spiritual beliefs have been found to play a significant role in domestic violence dynamics. Certain religious interpretations and teachings can contribute to the acceptance of violence, particularly against women, as a form of submission or obedience. This phenomenon is prevalent in Middle Eastern countries, where religious texts such as the Bible and the Qur’an are often quoted to justify and perpetuate gender-based violence (20).

For example, in the book of Ephesians 5:22–24, the Bible states that wives should submit themselves to their husbands, equating the husband’s authority to that of the Lord. Similarly, the Qur’an emphasizes the importance of wives being sexually available to their husbands in all aspects of their relationship. These religious teachings can create a belief system where women are expected to endure mistreatment and forgive their abusive partners (15).

The influence of religious beliefs and practices can complicate a woman’s decision to leave an abusive relationship, particularly when marriage is considered a sacred institution. Feelings of guilt and difficulties in seeking support or ending the relationship can arise due to the belief that marriage is ordained by God (15).

It is important to note that the response of religious congregations and communities to domestic violence can vary. In some cases, if abuse is ignored or not condemned, it may perpetuate the cycle of violence and hinder efforts to support victims and hold perpetrators accountable. However, in other instances, religious organizations may provide emotional support and assistance through dedicated sessions aimed at helping all affected family members heal and address the violence (20).

Recognizing the influence of religious beliefs on domestic violence is crucial for developing comprehensive interventions and support systems that address the specific challenges faced by individuals within religious contexts. This includes promoting awareness, education, and dialog within religious communities to foster an understanding that violence is never acceptable and to facilitate a safe environment for victims to seek help and healing.

### 2.4. Presence of risk factor (B)

#### 2.4.1. Depression, anxiety, and stress (B1)

Within households, the occurrence of violence is unfortunately prevalent, often stemming from economic constraints, social and psychological problems, depression, and stress. These factors instill such fear in the victims that they are often hesitant to report the abuse to the authorities (42).

Notably, when women assume the role of heads of households, they experience significantly higher levels of depression compared to men (21). This study highlights that the presence of poverty, financial struggles, and the ensuing violence associated with these circumstances significantly elevate the risk of women experiencing severe health disorders, necessitating urgent prioritization of their well-being. Regrettably, in low-income countries where cases of depression are on the rise within public hospitals, the provision of adequate care becomes an insurmountable challenge (21).

These findings underscore the urgent need for comprehensive support systems and targeted interventions that address the multifaceted impact of domestic violence on individuals’ mental and physical health. Furthermore, effective policies should be implemented to alleviate economic hardships and provide accessible mental health services, particularly in low-income settings. By addressing the underlying factors contributing to violence within households and ensuring adequate care for those affected, society can take significant strides toward breaking the cycle of violence and promoting a safer and more supportive environment for individuals and families.

#### 2.4.2. Retention tendency (B2)

Many societies, particularly in Africa, are characterized by a deeply ingrained patriarchal social structure, where men hold the belief that they have the right to exert power and control over their partners (31). This ideology of patriarchy is often reinforced by women themselves, who may adhere to traditional gender roles and view marital abuse as a norm rather than recognizing it as an act of violence. This acceptance of abuse is influenced by societal expectations and cultural norms that prioritize the preservation of marriage and the submission of women.

Within these contexts, there is often a preference for male children over female children, as males are seen as essential for carrying on the family name and lineage (43). This preference is also reflected in the distribution of property and decision-making power within households, where males are given greater rights and authority. Such gender-based inequalities perpetuate the cycle of power imbalances and contribute to the normalization of violence against women.

It is important to note that men can also be victims of domestic violence. However, societal and cultural norms have long portrayed men as strong and superior figures, making it challenging for male victims to come forward and report their abusers due to the fear of being stigmatized and rejected by society (16). The cultural expectations surrounding masculinity create barriers for men seeking help and support, further perpetuating the silence around male victimization.

These cultural dynamics underscore the complexity of domestic violence within patriarchal societies. Challenging and dismantling deeply rooted gender norms and power structures is essential for addressing domestic violence effectively. This includes promoting gender equality, empowering women, and engaging men and boys in efforts to combat violence. It also requires creating safe spaces and support systems that encourage both women and men to break the silence, seek help, and challenge the harmful societal narratives that perpetuate violence and victim-blaming.

#### 2.4.3. Density (B3)

Moreover, the issue of overcrowding within households has emerged as another important factor influencing domestic violence. Overcrowding refers to the stress caused by the presence of a large number of individuals in a confined space, leading to a lack of control over one’s environment (44). This overcrowding can have a detrimental impact on the psychological well-being of household members, thereby negatively affecting their internal relationships.

The freedom to use spaces within the home and the ability to control interactions with others have been identified as crucial factors that contribute to satisfaction with the home environment and the way individuals relate to each other. In this regard, studies have shown that when households are crowded, and individuals lack personal space and control over their living conditions, the risk of violence may increase (45).

Furthermore, investigations conducted during periods of extensive confinement, such as the COVID-19 pandemic, have shed light on the significance of other environmental factors within homes (46). For instance, aspects like proper ventilation and adequate living space have been found to influence the overall quality of life and the health of household inhabitants.

These findings emphasize the importance of considering the physical living conditions and environmental factors within households when examining the dynamics of domestic violence. Addressing issues of overcrowding, promoting healthy and safe living environments, and ensuring access to basic amenities and resources are crucial steps in reducing the risk of violence and improving the well-being of individuals and families within their homes.

#### 2.4.4. Reason for confrontation (B4)

Another form of violence that exists within households is abandonment and neglect, which manifests through a lack of protection, insufficient physical care, neglecting emotional needs, and disregarding proper nutrition and medical care (47). This definition highlights that any member of the family can be subjected to this form of violence, underscoring the significance of recognizing its various manifestations.

In this complex context, negative thoughts and emotions can arise, leading to detrimental consequences. For instance, suspicions of infidelity and feelings of jealousy can contribute to a decrease in the partner’s self-esteem, ultimately triggering intimate partner violence that inflicts physical, social, and health damages (32, 48).

Furthermore, it is important to acknowledge the intimate connection between domestic violence and civil issues. Marital conflicts, particularly when accompanied by violence, whether physical or psychological, can lead to a profound crisis within the relationship, often resulting in divorce. Unfortunately, the process of obtaining a divorce or establishing parental arrangements can be protracted, creating additional friction and potentially exacerbating gender-based violence (49).

These dynamics underscore the complex interplay between domestic violence and broader social, emotional, and legal contexts. Understanding these interconnected factors is crucial for developing effective interventions and support systems that address the multifaceted nature of domestic violence, promote healthy relationships, and safeguard the well-being of individuals and families within the home.

Finally, despite the multitude of factors identified in the existing literature that may have an impact on gender-based violence, we have selected a subset of variables for our study based on data availability. Specifically, our analysis will concentrate on the following factors reviewed: (A3) religion, (B1) depression, health consciousness, and mood, (B2) retention tendency as reflected by household chores, and (B3) density.

The rationale behind our choice of these variables stems from their perceived significance and potential relevance to the study of domestic violence. Religion has been widely acknowledged as a social and cultural determinant that shapes beliefs, values, and gender roles within a society, which may have implications for power dynamics and relationship dynamics within households. Depression, as a psychological construct, has been frequently associated with increased vulnerability and impaired coping mechanisms, potentially contributing to the occurrence or perpetuation of domestic violence. Health consciousness and mood are additional constructs that have garnered attention in the context of interpersonal relationships. Health consciousness relates to individuals’ awareness and concern for their own well-being and that of others, which may influence their attitudes and behaviors within the household. Mood, on the other hand, reflects emotional states that can influence communication, conflict resolution, and overall dynamics within intimate relationships.

Furthermore, we have included the variable of retention tendency, as manifested through household chores. This variable is indicative of individuals’ willingness or inclination to maintain their involvement and responsibilities within the household. It is hypothesized that individuals with higher retention tendencies may exhibit a greater commitment to the relationship, which could influence the occurrence and dynamics of domestic violence. Lastly, we consider the variable of density, which captures the population density within the living environment. This variable may serve as a proxy for socio-environmental conditions, such as overcrowding or limited personal space, which can potentially contribute to stress, conflict, and interpersonal tensions within households.

By examining these selected factors, we aim to gain insights into their relationships with domestic violence and contribute to a better understanding of the complex dynamics underlying such occurrences. It is important to note that these variables represent only a subset of the broader range of factors that influence gender-based violence, and further research is warranted to explore additional dimensions and interactions within this multifaceted issue.

## 3. Data collection and variables

The reference population for this study is Ecuadorian habitants. Participants were invited to fill up a survey concerning COVID-19 impact on their mental health. Data collection took place between April and May 2020, exactly at the time of the mandatory lockdowns taking place. In this context governmental authorities ordered mobility restrictions as well as social distancing measures. We conduct three waves of social media invitations to participate in the study. Invitations were sent using the institutional accounts of the universities the authors of this study are affiliated. At the end, we received 2,403 answers, 50.5% females and 49.5% males. 49% of them have college degrees.

### 3.1. Ecuador stylized facts

Ecuador, a small developing country in South America, has a population of approximately 17 million inhabitants, with a population density of 61.85 people per square kilometer.

During the months under investigation, the Central Bank of Ecuador reported that the country’s GDP in the fourth quarter of 2020 amounted to $16,500 million. This represented a decrease of 7.2% compared to the same period in 2019, and a 5.6% decline in the first quarter of 2021 compared to the same quarter of the previous year. However, despite these declines, there was a slight growth of 0.6% in the GDP during the fourth quarter of 2020 and 0.7% in the first quarter of 2021 when compared to the previous quarter.

In mid-March, the Ecuadorian government implemented a mandatory lockdown that lasted for several weeks. By July 30, 2020, Ecuador had reported over 80,000 confirmed cases of COVID-19. The statistics on the impact of the pandemic revealed a death rate of 23.9 per 100,000 inhabitants, ranking Ecuador fourth globally behind the UK, Italy, and the USA, with rates of 63.7, 57.1, and 36.2, respectively. Additionally, Ecuador’s observed case-fatality ratio stood at 8.3%, placing it fourth globally after Italy, the UK, and Mexico, with rates of 14.5, 14, and 11.9%, respectively (50). As the lockdown measures continued, mental health issues began to emerge among the population (51).

The challenging socioeconomic conditions and the impact of the pandemic on public health have had significant repercussions in Ecuador, highlighting the need for comprehensive strategies to address both the immediate and long-term consequences on the well-being of its population.

### 3.2. Dependent variable

The dependent variable in this study is Domestic Violence, which is measured using a composite score derived from five items. These items were rated on a 7-point scale, ranging from 1 (never) to 7 (very frequent), to assess the frequency of intrafamily conflict and violence occurring within the respondents’ homes. The five items included the following statements: “In my house, subjects are discussed with relative calm”; “In my house, heated discussions are common but without shouting at each other”; “Anger is common in my house, and I refuse to talk to others”; “In my house, there is the threat that someone will hit or throw something”; and “In my house, family members get easily irritated.”

To evaluate the internal consistency of the measurement, Cronbach’s Alpha was calculated and found to be 0.7. This indicates good internal consistency, suggesting that the items in the scale are measuring a similar construct and can be considered reliable for assessing the level of domestic violence within the households under investigation.

### 3.3. Independent variables

#### 3.3.1. Mood

The mood construct, based on Peterson and Sauber (52), is measured using three Likert scale questions. The respondents rate their agreement on a scale from strongly disagree to strongly agree. The questions included: “I am in a good mood,” “I feel happy,” and “At this moment, I feel nervous or irritable.” The Cronbach’s Alpha coefficient for this construct is 0.7757, indicating good internal consistency.

#### 3.3.2. Depression

The depression construct, based on the manual for the Depression Anxiety Stress Scales by Lovibond S and Lovibond P, is measured by summing the results of 13 Likert scale questions. The scale ranges from strongly disagreeing to strongly agreeing. The questions include: “I feel that life is meaningless,” “I do not feel enthusiastic about anything,” “I feel downhearted and sad,” and others. The Cronbach’s Alpha coefficient for this construct is 0.9031, indicating high internal consistency.

#### 3.3.3. Health consciousness

The health consciousness construct, based on Gould (53), is measured using four Likert scale questions. The respondents rate their agreement on a scale from strongly disagree to strongly agree. The questions include: “I’m alert to changes in my health,” “I am concerned about the health of others,” “Throughout the day, I am aware of what foods are best for my health,” and “I notice how I lose energy as the day goes by.” The Cronbach’s Alpha coefficient for this construct is 0.7, indicating acceptable internal consistency.

#### 3.3.4. Household chores

The respondents were asked to rate their involvement in various household chores on a scale from “not at all” to “a lot.” The listed household chores include cooking, washing dishes, cleaning restrooms, doing laundry, home maintenance, and helping with children/siblings. It can serve as a proxy for Retention Tendency.

#### 3.3.5. Density

It is measured as the number of people per bedroom, indicating the level of overcrowding within households.

#### 3.3.6. Religion

The religion construct is measured as the sum of four Likert scale items based on Worthington et al. (54). The respondents rate their agreement on a scale from strongly disagree to strongly agree. The items include: “My religious beliefs lie behind my whole approach to life,” “It is important to me to spend periods in private religious thought and reflection,” “Religion is very important to me because it answers many questions about the meaning of life,” and “I am informed about my local religious group and have some influence in its decisions.” The Cronbach’s Alpha coefficient for this construct is 0.8703, indicating good internal consistency.

### 3.4. Control variables

#### 3.4.1. Social media

The respondents were asked to indicate the number of hours they spend on social networks during a typical day. The scale ranges from “I do not review information on social networks” to “More than three hours.”

#### 3.4.2. Sex

Sex is measured as a binary variable, where 1 represents female and 0 represents male.

#### 3.4.3. Age

Age refers to the age of the respondent.

#### 3.4.4. Age of householder

Age of householder refers to the age of the individual who is the primary occupant or head of the household.

### 3.5. Describe statistics

Table 2 reports the means, standard deviation, and correlation matrix. Our dataset has not the presence of missing values.

**Table 2:** Summary statistics.

		Mean	SD	1	2	3	4	5	6	7	8	9	10
1	D. Violence	10.17603	3.10559	1									
2	Mood	13.3866	3.966022	−0.3045*	1								
3	*Depression*	38.5335	15.83018	0.3774*	−0.6162*	1							
4	*Health Cons*	20.81981	4.312471	−0.0545*	−0.0516*	0.1795*	1						
5	Social media	2.383271	1.137874	0.1200*	−0.1055*	0.1712*	0.0197	1					
6	Household chores	16.58843	6.621124	−0.0594*	0.0265	0.0181	0.2099*	−0.034	1				
7	Density	1.448138	0.7631484	0.1309*	−0.0796*	0.1085*	0.032	0.0083	0.0814*	1			
8	Religion	13.58177	6.810956	0.0104	0.0633*	0.0231	0.2335*	−0.0422*	0.1517*	0.0822*	1		
9	Age	30.69247	10.24113	−0.2046*	0.1058*	−0.1606*	0.1081*	−0.1259*	0.1080*	−0.1407*	0.1295*	1	
10	Age householder	48.46692	12.29038	0.0319	0.0408*	−0.0687*	0.0064	0.0478*	−0.1317*	−0.0235	0.0236	0.0981*	1

*p < 0.01.

Descriptive statistics reveal that the variables in the sample exhibit a considerable degree of homogeneity, as evidenced by the means being larger than the standard deviations. Moreover, the strong correlation between Depression and mood suggests that these two variables should not be included together in the same model.

## 4. Methodological approach

Our empirical identification strategy comprises the following linear model:


$$\begin{array}{l} Violence_{i}=\beta_{0}+\beta_{1}Mood_{i}+\beta_{2}Depression_{i}+\beta_{3}HealthCons_{i}\\ +\beta_{4}Socialmedia_{i}+\beta_{5}Householdchores_{i}+\beta_{6}Density_{i}\\ +\beta_{7}Religion_{i}+\beta_{8}Sex_{i}+\beta_{9}Age_{i}+\beta_{10}Age^{2}{}_{i}\\ +\beta_{11}Age\;householder_{i}+\beta_{12}Age\;householder^{2}+e_{i} \end{array}$$

We employed ordinary least squares (OLS) regression techniques to examine the relationship between our selected exogenous variables and household violence during the period of mandatory lockdowns. To ensure the robustness of our regression model, we conducted several diagnostic tests. Firstly, we tested for heteroscedasticity using the Breusch-Pagan test, yielding a chi-square value of 223.58 with a value of *p* of 0, indicating the presence of heteroscedasticity in the model. Secondly, we assessed multicollinearity using the variance inflation factor (VIF), which yielded a VIF value of 1.07, indicating no significant multicollinearity issues among the variables. Furthermore, we conducted the Ramsey Reset test to examine the presence of omitted variables in the model. The test yielded an F-statistic of 2.06 with a value of *p* of 0.103, suggesting no strong evidence of omitted variables. Lastly, we checked the normality of the residuals using the skewness and kurtosis tests, which yielded a chi-square value of 97.9 with a value of *p* of 0, indicating departure from normality in the residuals.

Hence, our analysis revealed the presence of heteroscedasticity issues and non-normality in the residuals. Consequently, it is imperative to employ an alternative estimation technique that can handle these challenges robustly. In light of these circumstances, we opted for Quantile Regression, as proposed by Koenker and Bassett (55), which allows for a comprehensive characterization of the relationship between the input variable(s) *x* and the dependent variable *y.*

### 4.1. Quantile regression

While an OLS predicts the average relationship between the independent variables and the dependent variable, which can cause the estimate to be unrepresentative of the entire distribution of the dependent variable if it is not identically distributed, Quantile Regression allows estimating parts of the dependent variable. Distribution of the dependent variable and thus determine the variations of the effect produced by the exogenous variables on the endogenous variable in different quantiles (56). The Quantile Regression methodology also presents the benefit that, by providing them with a weight, the errors are minimal. Quantile Regression is defined as follows:


$$Y_{i}=X_{i}\beta \left(\vartheta \right)+e_{\vartheta i}$$

$$=Q_{\vartheta }\left(Y_{i}\right)+e_{\vartheta i},0<\vartheta <1$$

where: $Y_{i}$ is dependent variable, $X_{i}$ is vector of independent variables, β(ϑ): is vector of parameters to be estimated for a given quantile ϑ, $e_{\vartheta i}$ : is random disturbance corresponding to the quantile ϑ, $Q_{\vartheta }\left(Y_{i}\right)$ is qth quantile of the conditional distribution of $Y_{i}$ given the known vector of regressors $X_{i}$ .

The Quantile Regression model provides predictions of a specific quantile of the conditional distribution of the dependent variable and is considered the generalization of the sample quantile of an independent and identically distributed random variable (57). By considering a range of quantiles, Quantile Regression offers a more nuanced understanding of the conditional distribution, making it a valuable technique for analyzing various aspects of the relationship between variables.

## 5. Results

The estimation results are reported in Table 3. The regressions 1 and 3 consider individuals who are not household heads, while regressions 2 and 4 involve the respondent being the household head. In regressions 5 and 6, the respondent is not the household head and is also female, whereas in regressions 7 and 8, the respondents are household heads and male. The regressions exhibit a coefficient of determination ranging between 9 and 11.

**Table 3:** Results.

	(1)	(2)	(3)	(4)	(5)	(6)	(7)	(8)
	Violence	Violence	Violence	Violence	Violence	Violence	Violence	Violence
	Responder is not head of household	Responder is head of household	Responder is not head of household	Responder is head of household	Responder is not head of household (female)	Responder is head of household (female)	Responder is not head of household (male)	Responder is head of household (male)
Mood			−0.311*** [0.0271]	−0.206*** [0.0431]	−0.275*** [0.0395]	−0.265*** [0.0648]	−0.339*** [0.0381]	−0.178*** [0.0583]
Depression	0.0929*** [0.00664]	0.0703*** [0.0106]						
Health Consciousness	−0.0683*** [0.0244]	−0.140*** [0.0406]	−0.0481* [0.0253]	−0.149*** [0.0434]	−0.00663 [0.0366]	−0.0960 [0.0679]	−0.0644* [0.0360]	−0.177*** [0.0581]
Social media	0.146 [0.0892]	0.328** [0.144]	0.188* [0.092]	0.345** [0.155]	0.175 [0.133]	0.196 [0.234]	0.167 [0.135]	0.470** [0.210]
Household chores	−0.0236 [0.0159]	−0.00957 [0.0257]	−0.0396** [0.0167]	0.00336 [0.0280]	−0.0256 [0.0239]	0.0427 [0.0421]	−0.0610** [0.0244]	−0.0114 [0.0379]
Density	0.265* [0.136]	0.728*** [0.203]	0.198 [0.143]	0.638*** [0.219]	0.323* [0.195]	0.513 [0.311]	0.0399 [0.222]	0.538* [0.306]
Religion	0.0178 [0.0155]	0.0710*** [0.0233]	0.0302* [0.0164]	0.0782*** [0.0253]	0.00485 [0.0232]	0.0727* [0.0393]	0.0568** [0.0238]	0.0745** [0.0339]
Sex	−0.00513 [0.213]	−0.0225 [0.352]	0.180 [0.224]	0.164 [0.381]				
Age	−0.0854 [0.0607]	−0.0641 [0.102]	−0.135 [0.0634]	−0.0844 [0.109]	−0.114 [0.0847]	0.0541 [0.188]	−0.159 [0.119]	−0.171 [0.139]
Age^2^	0.000573 [0.000849]	0.000743 [0.00113]	0.00110 [0.000889]	0.000675 [0.00122]	0.000808 [0.00115]	−0.000443 [0.00214]	0.00158 [0.00183]	0.00148 [0.00154]
Age householder	−0.0520 [0.0613]		0.0427 [0.0643]		0.0523 [0.0893]		0.0115 [0.0992]	
Age householder^2^	0.000607 [0.000593]		−0.000389 [0.000623]		−0.000382 [0.000878]		−0.000174 [0.000942]	
_cons	12.14*** [2.056]	10.26*** [2.386]	18.23*** [2.167]	16.50*** [2.573]	16.15*** [2.994]	12.49*** [4.323]	20.58*** [3.339]	18.91*** [3.305]
*N*	1802	597	1802	597	992	195	810	402
Pseudo R^2^	0.1090	0.1161	0.0878	0.0869	0.0906	0.1136	0.0916	0.0960

Standard errors in brackets. *p < 0.1, ** *p* < 0.05, *** *p* < 0.001.

The effects of the different variables studied on violence are presented below: Across all regressions, it can be observed that the mood of a person, which indicates whether they are in a good mood or feeling cheerful, nervous, or irritated, is statistically significant at all levels of confidence. This implies that violence decreases when the mood is good. On the other hand, depression has a positive and significant sign. This tells us that, on average, an increase of one unit in the depression, anxiety, and stress scale is associated with an increase in the measurement of conflict and intrafamily violence in a household, whether the respondent is a household head or not.

On the other hand, Health Consciousness has a negative and significant sign, indicating that violence decreases as Health Consciousness increases. However, it is noteworthy that it loses significance when the survey respondent is a woman, regardless of whether she is a household head or not.

Regarding Household chores, which refers to the time spent on household tasks, it can be observed that it is only significant and negative when the respondent is not a household head, and this significance holds even when the respondent is male. In other words, less time spent on household chores decreases violence in households where the respondent is not a household head.

The variable religion generally has a positive and significant sign in most regressions, but loses significance in regressions (1) and (5), where the respondent is not the household head and is female, respectively. This suggests that being religious would increase the levels of violence.

In general, density increases violence in the surveyed households, as indicated by a positive and significant sign. However, it is interesting to note that it is only significant again when the respondent is not a household head and is female, or when the respondent is a household head and is male.

As for the control variables, the variable Social media, which indicates the number of hours a person spends on social media, is positive and significant whether the respondent is a household head or not, and even when the respondent is male. This suggests that violence decreases with access to social media, possibly due to increased access to information. Finally, the variables sex, age of the respondent, and age of the household head were not significant.

## 6. Discussion

Interestingly, the prevalence and intensity of domestic violence appear to vary across different segments of society. Goodman (33) have highlighted the existence of variations in episodes of domestic violence among social strata. They have also identified several factors that act as deterrents to domestic violence, including income levels, educational attainment, employment status of the household head, household density, consumption of psychotropic substances, anxiety, and stress. These factors increase the likelihood of experiencing instances of violence within the home.

Within this context, the COVID-19 pandemic has had far-reaching implications for individuals and families worldwide, with significant impacts on various aspects of daily life, including domestic dynamics. This study explores the relationship between household factors and violence within the context of the pandemic, shedding light on the unique challenges and dynamics that have emerged during this period.

Our findings highlight the importance of considering mental well-being in the context of domestic violence during the pandemic. We observe that positive mood is associated with a decrease in violence levels within households. This suggests that maintaining good mental health and emotional well-being during times of crisis can serve as a protective factor against violence. With the increased stress and anxiety caused by the pandemic, policymakers and practitioners should prioritize mental health support and interventions to address potential escalations in violence within households.

Furthermore, our results indicate that depression exhibits a positive association with violence. As individuals grapple with the impacts of the pandemic, such as job loss, financial strain, and social isolation, the prevalence of depression may increase. This finding underscores the urgent need for accessible mental health resources and support networks to address the heightened risk of violence stemming from increased levels of depression.

The study also reveals that health consciousness plays a crucial role in reducing violence within households. As individuals become more aware of the importance of maintaining their health amidst the pandemic, violence levels decrease. This suggests that promoting health awareness and encouraging healthy lifestyle choices can serve as protective factors against domestic violence. Public health initiatives and educational campaigns aimed at fostering health-conscious behaviors should be emphasized as part of comprehensive violence prevention strategies.

Interestingly, our analysis uncovers a mitigating effect of social media usage on violence levels during the pandemic. With the increased reliance on digital platforms for communication and information sharing, access to social media may provide individuals with alternative channels for expression and support, ultimately reducing the likelihood of violence. Recognizing the potential benefits of social media, policymakers and practitioners should explore ways to leverage these platforms to disseminate violence prevention resources, provide support, and promote positive social connections within households.

Additionally, our findings highlight the role of household chores and density in shaping violence levels during the pandemic. Less time spent on household chores is associated with decreased violence, indicating that redistributing domestic responsibilities may alleviate tension and conflict within households. The COVID-19 pandemic has disrupted routines and added new challenges to household dynamics, making it essential to consider strategies that promote equitable distribution of chores and support mechanisms for individuals and families.

Moreover, the positive association between household density and violence emphasizes the impact of living conditions during the pandemic. With prolonged periods of confinement and restricted mobility, crowded living spaces may intensify conflicts and escalate violence. Policymakers should prioritize initiatives that address housing conditions, promote safe and adequate living environments, and provide resources to mitigate the negative effects of overcrowding.

In this line, our study delves into the intricate relationship between household factors and violence during the COVID-19 pandemic, primarily within our specific context. However, it is valuable to consider how our findings align or diverge when juxtaposed with research from developed countries, where economic, social, and healthcare systems are typically more advanced. In developed countries, the impact of crises, such as the pandemic, could manifest differently due to varying levels of financial stability, access to support networks, and well-established healthcare systems.

For instance, while we observe that maintaining mental well-being serves as a protective factor against violence, developed countries might have better access to mental health resources and support networks, potentially magnifying the impact of positive mental health on violence prevention (58). Similarly, the positive association between health consciousness and reduced violence levels could be influenced by different perceptions of health and well-being in developed countries, where health awareness campaigns are more prevalent (51).

The mitigating effect of social media on violence levels during the pandemic might also vary across contexts. Developed countries might have more widespread and equitable access to digital platforms, leading to a stronger impact on violence reduction through alternative channels for communication and support (59). Conversely, regions with limited digital infrastructure could experience a smaller effect.

Additionally, comparing the role of religious affiliation and its influence on violence with findings from developed countries could reveal cultural variations in the interplay between religious teachings, gender dynamics, and violence (60). While our study suggests the need for interventions promoting peaceful religious interpretations, it is crucial to examine whether similar efforts have been successful in developed nations with distinct cultural norms and religious landscapes.

In this context, this study makes a significant contribution to the field of gender-based violence research by intricately examining the intersection of diverse socio-economic and psychological factors within the backdrop of the COVID-19 pandemic. The uniqueness of this article lies in its holistic approach to comprehend domestic violence dynamics amidst a global crisis. By dissecting and analyzing how mental health, health awareness, social media utilization, household chore distribution, living space density, and religious affiliation interact to influence violence levels, this study provides a deeper and nuanced insight into the factors contributing to the manifestation and prevention of gender-based violence. Moreover, by pinpointing areas where traditional gender norms and religious beliefs might exacerbate violence, the article suggests novel avenues for research and intervention development that account for cultural and contextual complexities. Ultimately, this work not only advances the understanding of gender-based violence during a critical period but also offers practical and theoretical recommendations to inform policies and preventive actions both throughout the pandemic and in potential future crises.

In considering the limitations of our study, we acknowledge that while our findings provide crucial insights into the role of religious affiliation in shaping violence levels during the pandemic, there are certain aspects that warrant further investigation. Firstly, our analysis primarily focuses on the association between religious beliefs and violence without delving deeply into the underlying mechanisms that drive this relationship. Future research could employ qualitative methodologies to explore how specific religious doctrines and practices interact with broader cultural norms to influence gender dynamics and contribute to violence within households. Additionally, our study does not extensively address variations in religious interpretations across different communities, which could lead to distinct outcomes in terms of violence prevention efforts. To address these limitations, scholars could conduct comparative studies across religious affiliations and denominations to uncover nuanced insights into the interplay between religious teachings, cultural contexts, and violence dynamics.

Furthermore, while our study suggests that policymakers and practitioners should consider developing targeted interventions promoting peaceful religious interpretations to mitigate violence, the precise design and effectiveness of such interventions remain areas ripe for exploration. Future research could involve collaboration with religious leaders and communities to develop and test intervention strategies that align with both religious teachings and contemporary gender equality principles. This interdisciplinary approach could yield actionable insights into fostering cultural change and enhancing the role of religion in promoting non-violence within households.

In conclusion, our study provides valuable insights into the dynamics of domestic violence within households during the COVID-19 pandemic. The findings underscore the importance of addressing mental health, promoting health consciousness, leveraging social media, redistributing household chores, improving housing conditions, and considering the nuanced role of religious beliefs. By incorporating these findings into policy and intervention strategies, policymakers and practitioners can work toward preventing and mitigating domestic violence in the context of the ongoing pandemic.

## Data availability statement

The raw data supporting the conclusions of this article will be made available by the authors, without undue reservation.

## Author contributions

CL played a crucial role in this research project, being responsible for the data collection, conducting the econometric analysis, contributing to the literature review, introduction, and discussion sections of the manuscript. JD-S made significant contributions to the project and assisted in the data collection process, contributed to the literature review, and provided insights in the discussion section. FV assisted with the data collection process and reviewed the article for accuracy and clarity. All authors contributed to the article and approved the submitted version.

## Funding

This project receives funding from Vicerrectorado de Investigación y Proyección Social, Escuela Politécnica Nacional.

## Conflict of interest

The authors declare that the research was conducted in the absence of any commercial or financial relationships that could be construed as a potential conflict of interest.

## Publisher’s note

All claims expressed in this article are solely those of the authors and do not necessarily represent those of their affiliated organizations, or those of the publisher, the editors and the reviewers. Any product that may be evaluated in this article, or claim that may be made by its manufacturer, is not guaranteed or endorsed by the publisher.

## References

[1] World Health Organization. (2021). Violence against women. Available at: https://www.who.int/es/news-room/fact-sheets/detail/violence-against-women

[2] Malik Sana Naeem K. (2020). Impact of COVID-19 pandemic on women: health, livelihoods & domestic violence. Sustainable Development Policy Institute. Available at: https://www.iassw-aiets.org/wp-content/uploads/2021/01/Covid19-impact-on-women.pdf

[3] Manrique De Lara A Arellano MDJM. The COVID-19 pandemic and ethics in Mexico through a gender lens. J Bioethical Inquiry. (2020) 17:613–17. doi: 10.1007/s11673-020-10029-4 32840852 PMC7445801

[4] Taub A. A new COVID-19 crisis: Domestic abuse rises worldwide. New York, NY: New York Times (2020).

[5] World Health Organization (2013). World health statistics 2013. Available at: https://www.who.int/publications/i/item/9789241564588

[6] Sepehrdoust H. Health care analysis and regional disparities in different provinces of Iran. Iran. Econ. Rev. (2009) 14:113–34.

[7] White JW Bondurant B. Gendered violence. Gendered relationships. (1996):197–210.

[8] Tan SY Haining R. Crime victimization and the implications for individual health and wellbeing: a Sheffield case study. Soc Sci Med. (2016) 167:128–39. doi: 10.1016/j.socscimed.2016.08.018 27619756

[9] Alvarez-Hernandez LR Cardenas I Bloom A. COVID-19 pandemic and intimate partner violence: an analysis of help-seeking messages in the Spanish-speaking media. J Fam Violence. (2021) 37:939–50. doi: 10.1007/s10896-021-00263-8 33678949 PMC7914115

[10] Moorer O. (2021). Intimate partner violence vs. domestic violence–YWCA Spokane. Ywca Spokane Organization. Available at: https://ywcaspokane.org/What-Is-Intimate-Partner-Domestic-Violence/

[11] Carmo R Grams A Magalhães T. Men as victims of intimate partner violence. J Forensic Legal Med. (2011) 18:355–9. doi: 10.1016/j.jflm.2011.07.006 22018167

[12] McTavish JR MacGregor JCD Wathen CN MacMillan HL. Children’s exposure to intimate partner violence: an overview. Int Rev Psychiatry. (2016) 28:504–18. doi: 10.1080/09540261.2016.1205001 27414209

[13] Wathen CN MacMillan HL. Children’s exposure to intimate partner violence: impacts and interventions. Paediatr Child Health. (2013) 18:419–22. doi: 10.1093/pch/18.8.419 24426794 PMC3887080

[14] Erten B Keskin P. For better or for worse? Education and the prevalence of domestic violence in Turkey. Am Econ J Appl Econ. (2018) 10:64–105. doi: 10.1257/app.20160278

[15] Krob DB Steffen L. Religious influence on education and culture: violence against women as common sense. Procedia Soc Behav Sci. (2015) 174:2374–9. doi: 10.1016/j.sbspro.2015.01.903

[16] Visaria L. Violence against women in India: is empowerment a protective factor? Econ Polit Wkly. (2008) 43:60–6. doi: 10.1007/s40609-020-00186-0

[17] Alonso-Borrego C Carrasco R. Employment and the risk of domestic violence: does the Breadwinner’s gender matter? SSRN Electron J. (2016) 49:5074–91. doi: 10.2139/ssrn.2748885

[18] Anderberg D Rainer H Wadsworth J Wilson T. Unemployment and domestic violence: theory and evidence. Econ J. (2016) 126:1947–79. doi: 10.1111/ecoj.12246

[19] Sen P. Enhancing women’s choices in responding to domestic violence in Calcutta: a comparison of employment and education. Eur J Dev Res. (1999) 11:65–86. doi: 10.1080/09578819908426739

[20] Tomisin A. Culture, religion and help-seeking for intimate partner violence victims in Nigeria (a narrative review). African J Soc Sci Human Res. (2020) 3:56.

[21] Zeybek O Arslan Examining cultural heritages harmed by religious fanaticism: sample of the Palmyra Ancient City. Bartın Orman Fakültesi Dergisi. (2017) 19:1–10.

[22] Van de Velde S Bracke P Levecque K. Gender differences in depression in 23 European countries. Cross-national variation in the gender gap in depression. Soc Sci Med. (2010) 71:305–13. doi: 10.1016/j.socscimed.2010.03.035 20483518

[23] Straus MA Gelles RJ Steinmetz SK. Behind closed doors: Violence in the American family Routledge (2017).

[24] Burney VH. Applications of social cognitive theory to gifted education. Roeper Review. (2008) 30:130–9.

[25] Cooper A Smith EL. Homicide trends in the United States, 1980–2008. Washington, DC: Bureau of Justice Statistics (2011).

[26] Heise L Garcia-Moreno C. Violence by intimate partners. World report on violence and health. (2002) 1:87–113.

[27] Langford L Isaac N Kabat S. Homicides related to intimate partner violence in Massachusetts: examining case ascertainment and validity of the SHR. Homicide Stud. (1998) 2:353–77. doi: 10.1177/1088767998002004002

[28] Walker-Descartes I Mineo M Condado LV Agrawal N. Domestic violence and its effects on women, children, and families. Pediatr Clin N Am. (2021) 68:455–64. doi: 10.1016/j.pcl.2020.12.011 33678299

[29] Ishola SA. Domestic violence: the Nigerian experience. Asia-Africa J Mission Ministry. (2016) 13:3–16. doi: 10.21806/aamm.2016.13.01

[30] Barrientos S Kothari U Phillips N. Dynamics of unfree labour in the contemporary global economy. J. Dev. Stud. (2013) 49:1037–41.

[31] Fareo D. Domestic violence against women in Nigeria. Europ J Psychol Res. (2015) 371:1664. doi: 10.1016/S0140-6736(08)60723-0

[32] Goodman L Epstein D. COVID-19 and the Loneliness Pandemic: Implications for Intimate Partner Violence Survivors (2020). Available at: 10.2139/ssrn.3727674 PMC7643096 33169047

[33] Babu BV Kar SK. Domestic violence against women in eastern India: a population-based study on prevalence and related issues. BMC Public Health. (2009) 9:1–15. doi: 10.1186/1471-2458-9-129 19426515 PMC2685379

[34] Semahegn A Mengistie B. Domestic violence against women and associated factors in Ethiopia; systematic review. Reprod Health. (2015) 12:72. doi: 10.1186/s12978-015-0072-1 26319026 PMC4553009

[35] Farley R. Homicide trends in the United States. Demography. (1975) 17:177–88. 7389959

[36] Owoaje ET OlaOlorun FM. Women at risk of physical intimate partner violence: a cross-sectional analysis of a low-income community in Southwest Nigeria. Afr J Reprod Health. (2012) 16:43–53. PMID: 22783667

[37] Bhalotra S Kambhampati U Rawlings S Siddique Z. Intimate partner violence: the influence of job opportunities for men and women. World Bank Econ Rev. (2021) 35:461–79. doi: 10.1093/wber/lhz030

[38] Kargar Jahromi M Jamali S Rahmanian Koshkaki A Javadpour S. Prevalence and risk factors of domestic violence against women by their husbands in Iran. Global J Health Sci. (2015) 8:175–83. doi: 10.5539/gjhs.v8n5p175 PMC4877196 26652083

[39] Sen S Bolsoy N. Violence against women: prevalence and risk factors in Turkish sample. BMC Womens Health. (2017) 17:1–9. doi: 10.1186/s12905-017-0454-3 29100515 PMC5670523

[40] Croda E Grossbard S. Women pay the price of COVID-19 more than men. Rev Econ Househ. (2021) 19:1–9. doi: 10.1007/s11150-021-09549-8 33613141 PMC7883943

[41] Tur-Prats A. Family types and intimate partner violence: a historical perspective. Rev Econ Stat. (2019) 101:878–91. doi: 10.1162/rest_a_00784

[42] Straus MA. Measuring intrafamily conflict and violence: the conflict tactics (CT) scales. Phys Violen Am Fam. (2019) 41:29–48. doi: 10.4324/9781315126401-4

[43] Hsu L Kaufmann P Srinivasan S. How do franchise ownership structure and strategic investment emphasis influence stock returns and risks? J Retail. (2017) 93:350–68. doi: 10.1016/j.jretai.2017.04.004

[44] Díaz-Sánchez JP Lanchimba C Velasco F Paz M. Associations between household density and mood during COVID-19 lockdowns: evidence from Ecuador associations between household density and mood during COVID-19 lockdowns: evidence from Ecuador. Cities Health. (2022):1–10. doi: 10.1080/23748834.2022.2135187

[45] Porter C Favara M Sánchez A Scott D. The impact of COVID-19 lockdowns on physical domestic violence: evidence from a list randomization experiment. SSM Populat Health. (2021) 14:792. doi: 10.1016/j.ssmph.2021.100792 PMC8080075 33948480

[46] Diaz A Nucci-Sack A Colon R Guillot M Hollman D Brunelli M. Impact of COVID-19 mitigation measures on Inner-City female youth in New York City. J Adolesc Health. (2022) 70:220–7. doi: 10.1016/j.jadohealth.2021.10.015 34836802 PMC8547169

[47] Barbara G Facchin F Micci L Rendiniello M Giulini P Cattaneo C. COVID-19, lockdown, and intimate partner violence: some data from an Italian service and suggestions for future approaches. J Women's Health. (2020) 29:1239–42. doi: 10.1089/jwh.2020.8590 33006492

[48] Piquero AR Riddell JR Bishopp SA Narvey C Reid JA Piquero NL. Staying home, staying safe? A short-term analysis of COVID-19 on Dallas domestic violence. Am J Crim Justice. (2020) 45:601–35. doi: 10.1007/s12103-020-09531-7 32837161 PMC7293590

[49] Lillard LA Waite LJ. A joint model of marital childbearing and marital disruption. Demography. (1993) 30:653–81. doi: 10.2307/2061812 8262286

[50] Johns Hopkins Coronavirus Resource Center. (2020). Mortality analyses. John Hopkins University & Medicine. Available at: https://coronavirus.jhu.edu/data/mortality

[51] Franklin V Cintya L Mariel PM Pablo DSJ. Mental health factors that guide individuals to engage in overconsumption behavior during the COVID-19 pandemic: a cross-cultural study between USA and Ecuador. Front Public Health. (2022) 10:1–12. doi: 10.3389/fpubh.2022.844947, PMID: 35392477 PMC8980352

[52] Peterson R Sauber M. “A mood scale for survey research.” In: Handbook of marketing scales, eds. H Briggs, A Howland, DE Axelsen, N Kabakian (Sage Publications, Inc.) (1983). 188.

[53] Gould SJ. Consumer attitudes toward health and health care: a differential perspective. J Consum Affairs. (1988) 22:96–118. doi: 10.1111/j.1745-6606.1988.tb00215.x

[54] Worthington EL Wade NG Hight TL Ripley JS McCullough ME Berry JW. The religious commitment Inventory-10: development, refinement, and validation of a brief scale for research and counseling. J Couns Psychol. (2003) 50:84–96. doi: 10.1037/0022-0167.50.1.84

[55] Koenker R Bassett G. Regression Quantiles. Econometrica. (1978) 46:33. doi: 10.2307/1913643

[56] Conyon MJ He L. Firm performance and boardroom gender diversity: a quantile regression approach. J Bus Res. (2017) 79:198–211. doi: 10.1016/J.JBUSRES.2017.02.006

[57] Dimelis S Louri H. Foreign ownership and production efficiency: a quantile regression analysis. Oxf Econ Pap. (2002) 54:449–69. doi: 10.1093/oep/54.3.449

[58] Betancourt TS Khan KT. The mental health of children affected by armed conflict: protective processes and pathways to resilience. Int Rev Psychiatry. (2008) 20:317–28. doi: 10.1080/09540260802090363 18569183 PMC2613765

[59] Mcdonald P Charlesworth S Graham T. Developing a framework of effective prevention and response strategies in workplace sexual harassment. Asia Pac J Hum Resour. (2015) 53:41–58. doi: 10.1111/1744-7941.12046

[60] Ellison CG Bartkowski JP Anderson KL. Are there religious variations in domestic violence? J Fam Issues. (1999) 20:87–113. doi: 10.1177/019251399020001005

